# Comparison of WBGTs over Different Surfaces within an Athletic Complex

**DOI:** 10.3390/medicina56060313

**Published:** 2020-06-25

**Authors:** Andrew Grundstein, Earl Cooper

**Affiliations:** 1 Department of Geography, University of Georgia, Athens, GA 30602, USA; 2 Department of Kinesiology, University of Georgia, Athens, GA 30602, USA; cooperb@uga.edu

**Keywords:** athletic surfaces, WBGT, weather, heat stress, safety

## Abstract

*Background and objectives*: Many athletic governing bodies are adopting on-site measurement of the wet-bulb globe temperature (WBGT) as part of their heat safety policies. It is well known, however, that microclimatic conditions can vary over different surface types and a question is whether more than one WBGT sensor is needed to accurately capture local environmental conditions. *Materials and Methods:* Our study collected matched WBGT data over three commonly used athletic surfaces (grass, artificial turf, and hardcourt tennis) across an athletic complex on the campus of the University of Georgia in Athens, GA. Data were collected every 10 min from 9:00 a.m. to 6:00 p.m. over a four-day period during July 2019. *Results*: Results indicate that there is no difference in WBGT among the three surfaces, even when considered over morning, midday, and afternoon practice periods. We did observe microclimatic differences in dry-bulb temperature and dewpoint temperature among the sites. Greater dry-bulb and lower dewpoint temperatures occurred over the tennis and artificial turf surfaces compared with the grass field because of reduced evapotranspiration and increase convective transfers of sensible heat over these surfaces. The lack of difference in WBGT among the surfaces is attributed to the counterbalancing influences of the different components that comprise the index. *Conclusions*: We conclude that, in a humid, subtropical climate over well-watered grass, there is no difference in WBGT among the three athletic surfaces and that, under these circumstances, a single monitoring site can provide representative WBGTs for nearby athletic surfaces.

## 1. Introduction

Exertional heat illnesses (EHI) affect thousands of athletes each year and exertional heat stroke is among the leading causes of death among athletes [1]. Environmental monitoring coupled with activity modification is a key component of a well-designed heat policy [2,3]. Importantly, on-site measurements can better capture local microclimate conditions than remote observations from weather stations as differences in sheltering, surface type, or solar exposure can influence heat stress [4,5,6]. As such, regarding the interscholastic participant, numerous high school athletic associations now require on-site measurement of environmental conditions using the wet-bulb globe temperature (WBGT) [7]. A question that has been raised among sports medicine professionals is whether a single weather measurement can represent environmental conditions on nearby athletic fields when there are a variety of surfaces (e.g., grass, artificial turf, hardcourt tennis, etc.) used for athletic play or if measurements are required over each surface. Microclimatic conditions over small areas can be greatly affected by the characteristics of the underlying surface [8]. Multiple studies have identified that athletic surface type, especially artificial turf, alter surface temperatures relative to grass covered surfaces, and which may affect heat stress [9,10,11,12,13]. What has been less explored is how these surface changes impact ambient air temperatures and humidity levels above the athletic surfaces and integrated bioclimatic indices like the WBGT [5,14,15,16]. Both the cost of WBGT sensors and the staffing to monitor multiple sensors may pose barriers to high schools or other organizations adopting the practice of monitoring environmental conditions where multiple sites are used. Our study seeks to identify if different athletic surfaces, which are commonly present on high school and college campuses, and other athletic/recreational facilities may affect WBGT measurements. In particular, we ask two key questions:

(1) Does the WBGT vary by athletic surface (artificial turf, hardcourt tennis, and grass)?
(2) Is a single monitoring station able to capture local WBGT conditions in an athletic complex?

## 2. Materials and Methods

**Figure 1:**
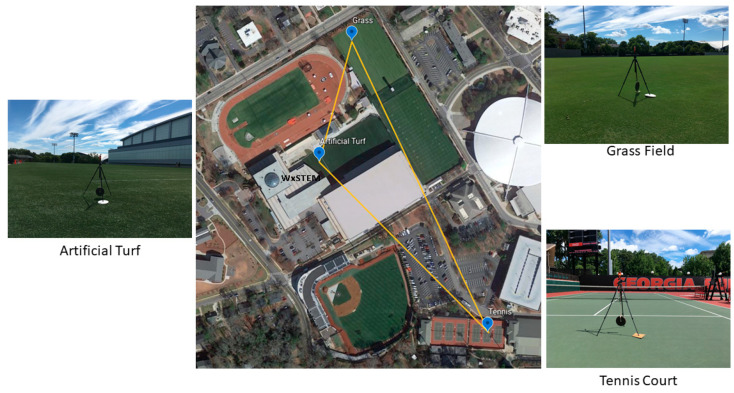
Map of study locations with site photographs. The distance between tennis and grass surfaces is 424 m, artificial turf and tennis surfaces is 317 m, and artificial turf and grass surfaces is 162 m. WxSTEM refers to the on-site weather station.

WBGT data were collected over three different surface types commonly associated with the sports of American football, soccer, and tennis on the campus of the University of Georgia (UGA) in Athens, GA, USA over a five-day period, 24–28 July, 2019 (Figure 1). Athens, GA has a humid, subtropical climate characterized by hot and humid summers [17]. Data were collected over commonly used athletic surfaces, including natural grass, artificial turf (FieldTurf, Montreal, QC, Canada), and hardcourt tennis (Plexipave, Andover, MA, USA) surfaces, which were all located between 162 and 423 m of each other. The natural grass surface was well watered. The day before the study (23 July), 3.56 mm of precipitation was recorded at the on-site WeatherSTEM station and the grass field was watered between 2:30 a.m. and 3:30 a.m. on two days (26 and 28 July) during the study. Three WBGT monitors (Kestrel 5400 heat stress meters, Nielsen-Kellerman, Boothwyn, PA, USA) were set on a tripod at each site in a sunny location that would not be subjected to shade (other than cloud cover) during the data collection period. In addition, the locations we selected were at least 15 m from another surface type and had sheltering that would reduce the effects of local advection. The tennis court, for instance, is located adjacent to an asphalt parking lot but is separated by a mesh covered fence that would reduce wind speeds.

Over each surface, the WBGT monitors were set up on the tripods at 1.2 m above the surface to represent an anthropometric scale [18]. The dry-bulb temperature, natural wet-bulb temperature, globe temperature, dewpoint temperature, and wind speed were collected every 10 min from 9:00 a.m. to 6:00 p.m. The WBGT was computed as a weighted average of the dry-bulb temperature (DB), natural wet-bulb temperature (WB), and globe temperature (GT) using the following equation [19]:

WBGT = 0.7 × WB + 0.2 × GT + 0.1 × DB.(1)

We followed the manufacturer’s recommendation and allowed the unit to equilibrate for 15 min prior to collecting data for analysis [20]. Observations with relative humidity <15% or >95% were removed from the dataset as they exceed the specification range for sensor accuracy [21]. For comparison purposes, only times when all three surfaces had viable observations were retained. In total, 243 observations from each surface were compared. These observations were divided into three different practice periods: morning (9:00–11:59 a.m., *n* = 63), midday (noon–2:59 p.m., *n* = 90), and afternoon (3:00–5:59 p.m., *n* = 90). Weather data (e.g., temperature, humidity, wind speed, and solar radiation) were collected from a WeatherSTEM station that is located within the sports complex (Figure 1). This weather dataset was used to identify the overall weather conditions during the study days [22].

Summary statistical measures were used to quantify WBGTs and other meteorological variables among the surfaces, with a focus on median for central tendency and interquartile range for variability as not all data distributions were normal. Normality was determined using the Kolmogorov–Smirnov test and visual inspection of the Q–Q plot and histogram. Pearson’s correlation coefficient was used to assess the association of WBGTs between the surfaces (i.e., grass vs. artificial turf; grass vs. tennis, and artificial turf vs. tennis) and the relationship between the WBGT over a particular surface with weather station data (e.g., temperature, dewpoint temperature, wind speed, and solar radiation). ANOVA (or Kruskal–Wallis one-way analysis of variance on ranks when the required assumptions were not met) was used to compare the effect of athletic surface type on WBGT values using α = 0.05. A similar approach was used to assess the effect of surface type on the WBGT components as well as dewpoint temperature. All statistical analyses were completed using SPSS (version 26; IMB Corp, Armonk, NY, USA).

## 3. Results

### 3.1. Weather Conditions

**Figure 2:**
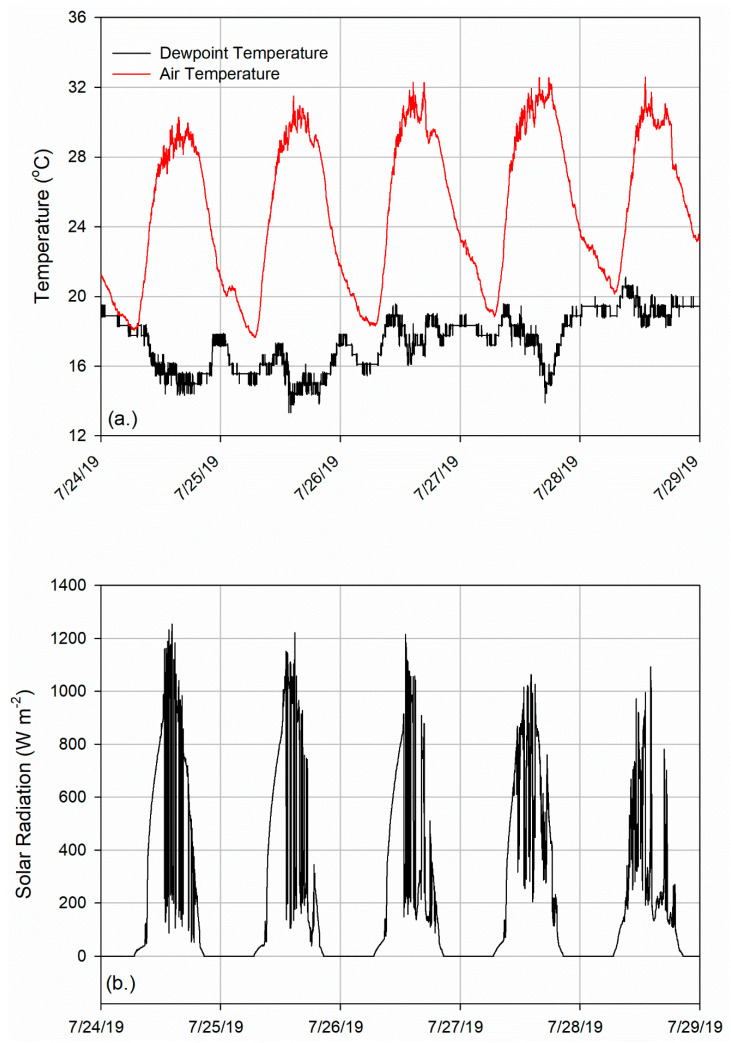
Weather conditions at on-site WeatherSTEM observing station: (**a**) air temperature and dewpoint temperature and (**b**) solar radiation.

Weather conditions were determined from a centrally located weather observing station (Figure 1). Over the five-day study, maximum air temperatures ranged from 30.3 to 32.6 °C and minimum temperatures were between 17.7 and 20.2 °C (Figure 2a). Average daily dewpoint temperature varied from 15.6 to 19.4 °C (Figure 2a). Maximum solar radiation exceeded 1000 W m^−2^ each day (1032–1097 W m^−2^) with considerable variability, particularly in the afternoon in response to changing cloud cover (Figure 2b). Based on 11 years (2009–2019) of July data from a nearby weather station at the UGA Climatology Research Laboratory (~1.8 km from study site with a longer period of record than the WeatherSTEM station), the study days had lower than average maximum daytime temperatures (long-term mean = 33.2 °C) and humidity (long-term mean = 22.5 °C) but peak solar radiation values that were close to average.

### 3.2. Differences in WBGT among Athletic Surfaces

**Figure 3:**
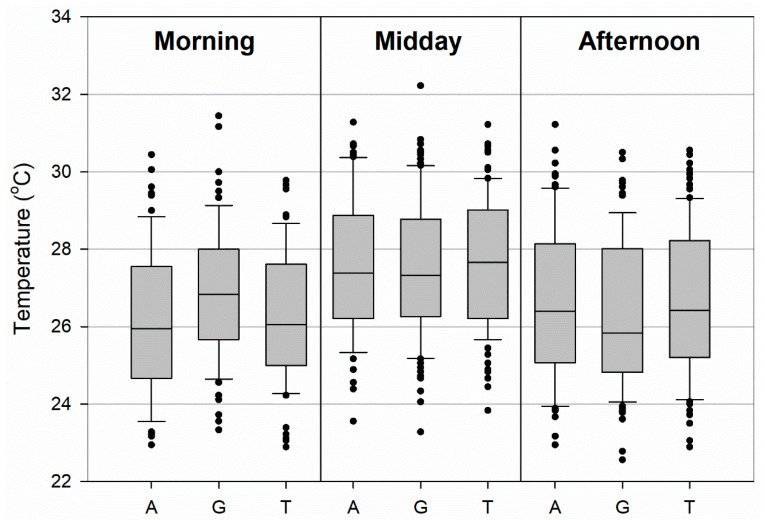
Box plots of wet-bulb globe temperature (WBGT) for artificial turf (A), grass (G), and hardcourt tennis (T) athletic surfaces for morning (9:00–11:59 a.m.), midday (noon–2:59 p.m.), and afternoon (3:00–5:59 p.m.) practice sessions. The boundaries of the box represent the 25th and 75th percentiles, the line within the box indicates the median, the whiskers are the 10th and 90th percentiles, and the points above and below are the outliers, respectively.

A wide variety of WBGTs occurred over the study period, ranging from as low at 22.9 °C up to 32.2 °C (Figure 3). In the morning period, median WBGTs ranged between 25.94 and 26.83 °C among the surfaces with median values slightly greater (0.78–0.89 °C) over grass than tennis or artificial turf surfaces, respectively. During the midday period, median WBGTs were greater relative to both the morning and afternoon practice times, with values ranging from 27.33 to 27.67 °C. This period had the smallest difference among median WBGTs, with artificial turf 0.06 °C greater and tennis 0.33 °C greater than grass. Finally, WBGTs decreased in the afternoon period relative to midday, with median values between 25.83 and 26.42 °C. Both artificial turf and tennis surfaces had slightly greater WBGTs than grass by approximately 0.56–0.58 °C. The afternoon had the largest variance of WBGTs values with the interquartile (75th–25th percentile) range from 3.01 to 3.19 °C compared with 2.33–2.89 °C for morning and 2.51–2.81 °C for midday. The athletic surface type did not have a significant effect on WBGT at the *p* < 0.05 level in any of the practice periods: F(2,186) = 2.828, *p* = 0.062 for morning, F(2,267) = 0.254, *p* = 0.776 for midday, and F(2,267) = 0.831, *p* = 0.437.

**Figure 4:**
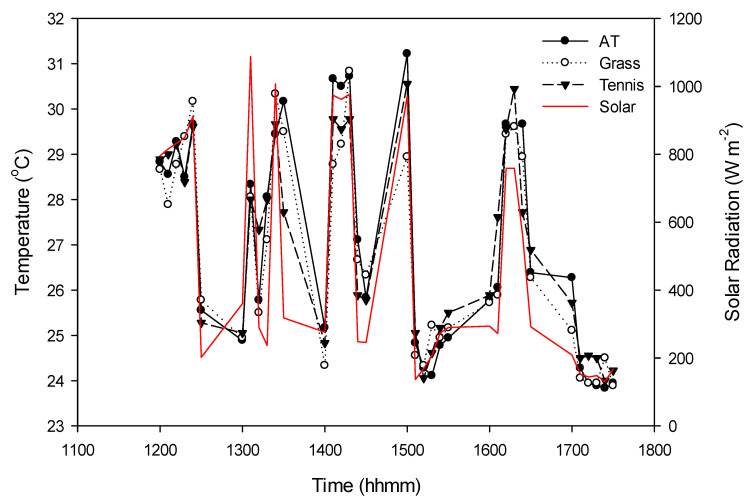
WBGTs and solar radiation by athletic surface for 26 July 2019 during the midday and afternoon periods. AT is artificial turf.

We observed strong correlations between the WBGTs of each surface, ranging from *r* = 0.89–0.92 in the morning, 0.81–0.90 during midday, and 0.90–0.93 in the afternoon. This is well illustrated in Figure 4 for 26 July between 11:00 a.m. and 5:59 p.m. where WBGTs over each surface type vary together in close association with recorded solar radiation levels. Of note are the large swings in WBGT by up to 5–6 °C in magnitude over short time periods (10 min) in response to changing solar radiation. In fact, over the study period, WBGTs were most highly correlated with changes in solar radiation (*r* = 0.60–0.66; Table 1). There were smaller correlations between WBGTs and air temperature (0.32–0.52) and dewpoint temperature (0.15–0.23).

### 3.3. Differences in Microclimates among Athletic Surfaces

**Figure 5:**
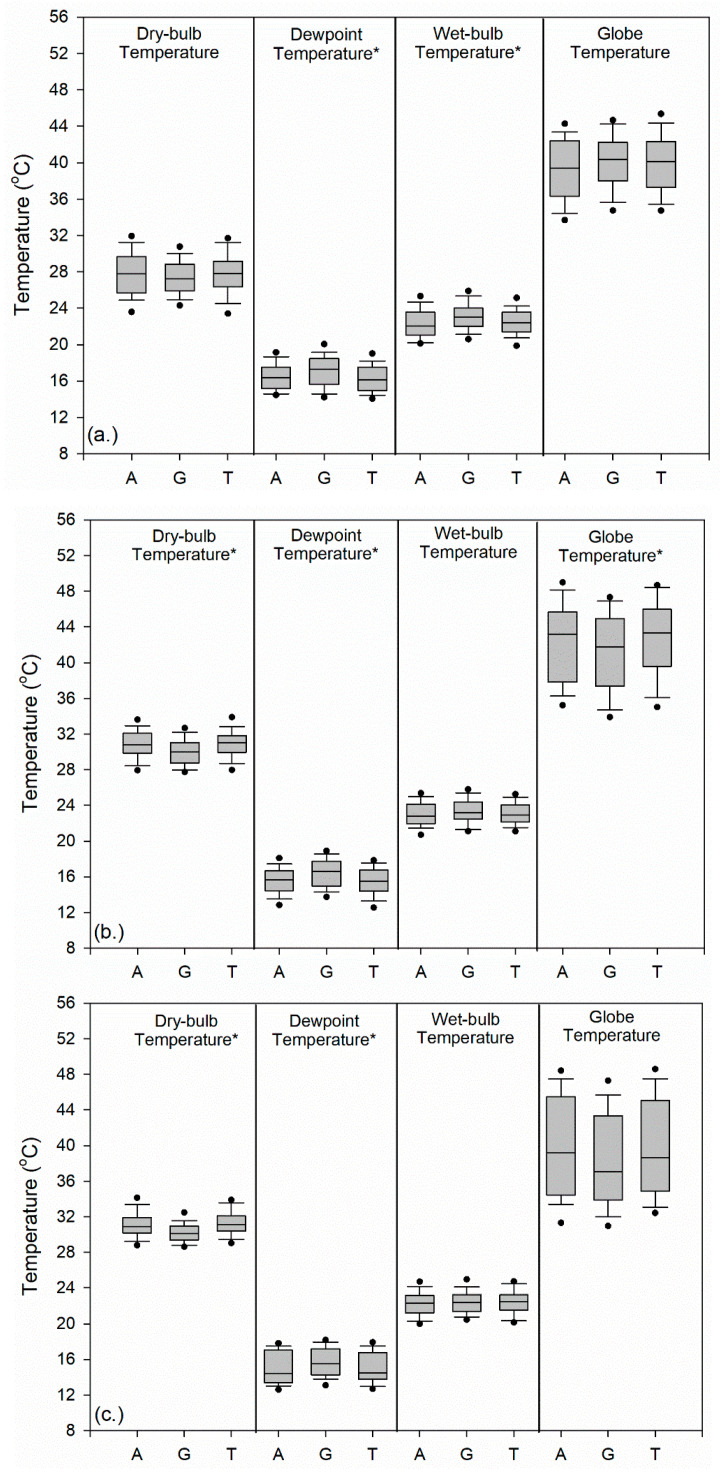
Box plots of WBGT components and other meteorological variables among artificial turf (A), grass (G), and hardcourt tennis (T) athletic surfaces for (**a**) morning (9:00–11:59 a.m.), (**b**) midday (noon–2:59 p.m.), and (**c**) afternoon (3:00–5:59 p.m.) practice sessions. The boundaries of the box represent the 25th and 75th percentiles, the line within the box indicates the median, the whiskers are the 10th and 90th percentiles, and the points above and below the whiskers are the 5th and 95th percentiles, respectively. Note that surface type had a statistically significant effect upon globe temperatures during midday but pairwise tests do not show any significant differences. * indicates statistically significant at *p* < 0.05.

We observed differences in the component parts of the WBGT as well as dewpoint temperature among the surfaces in the different time periods (Figure 5). In the morning, artificial turf and tennis have slightly warmer median dry-bulb temperatures (0.56–0.61 °C), but median dewpoints were 0.95–1.17 °C lower and wet-bulb temperatures were 0.61–0.94 °C lower than grass (Figure 5a). Median globe temperatures were greater over grass (+0.94 °C) and tennis (+0.72 °C) surfaces than artificial turf. The athletic surface type had a statistically significant effect on dewpoint temperature (F(2,186) = 3.583, *p* = 0.030). Post hoc comparisons using the Tukey HSD test indicated that the mean score was significantly different between grass and tennis surfaces (M = 0.732, *p* = 0.027). In addition, the surface type had a significant effect upon wet-bulb temperature (F(2,186) = 4.970, *p* = 0.008), with post hoc comparisons indicating that the mean value was significantly different between tennis and grass surfaces (M = −0.722, *p* = 0.022) and between artificial turf and grass surfaces (M = −0.7469, *p* = 0.017).

During midday, artificial turf and tennis surfaces had greater median dry-bulb temperatures by 0.83 to 1.06 °C, but dewpoints were 0.91 to 1.11 °C lower, and wet-bulb temperatures were 0.28 to 0.44 °C lower than measurements taken over grass. Median globe temperatures were greater (+1.42 to 1.53 °C) over the artificial turf and tennis court surfaces than the grass field (Figure 5b). The interquartile differences for the globe temperature over the three surfaces (approximately 6–8 °C) were greater than for dry-bulb, dewpoint, and wet-bulb temperatures (approximately 2–3 °C), indicating the greater dispersion of observations. Unlike in the morning, the athletic surface type had a statistically significant effect on dry-bulb temperatures (F(2,267) = 9.502, *p* = 0.000). Post hoc comparisons using the Tukey HSD test indicated that the mean dry-bulb temperature was significantly different between artificial turf and grass surfaces (M = 0.836, *p* = 0.001) and between tennis and grass (M = 0.934, *p* = 0.000). In addition, the surface type had a significant effect upon the dewpoint temperature (H(2) = 16.60, *p* = 0.000). Results from the pairwise tests using the Bonferroni correction show significant differences between tennis and grass (*p* = 0.001) and artificial turf and grass (*p* = 0.002) with respect to dewpoint measurements. Surface type had a significant effect upon globe temperatures (H(2) = 6.22, *p* = 0.045) but pairwise tests using the Bonferroni correction do not show any significant differences. This may have occurred because of the weakly significant global effect with the *p*-value near the 0.05 threshold.

Finally, during afternoon practices, artificial turf and tennis surfaces had greater median dry-bulb temperatures (0.81 to 1.00 °C) but lower dewpoint (1.06 to 1.09 °C lower) and wet-bulb temperatures (0.08 to 0.11 °C lower) than over grass (Figure 5c). Median globe temperatures were 1.61 to 2.19 °C greater over artificial turf and tennis surfaces than grass. The interquartile range for the globe temperature over the three surfaces (approximately 9–11 °C) are greater than for dry-bulb, dewpoint, and wet-bulb temperatures which are about 2–4 °C. Similar to midday, the athletic surface type had a statistically significant effect on dry-bulb temperatures (H(2) = 30.147, *p* = 0.000). Results from the pairwise tests using the Bonferroni correction show significant differences between artificial turf and grass (*p* = 0.000) and tennis and grass (*p* = 0.000) surfaces. The surface type also had a significant effect upon the dewpoint temperature (H(2) = 9.486, *p* = 0.009). Results from the pairwise tests using the Bonferroni correction show significant differences in dewpoint temperature between artificial turf and grass (*p* = 0.015) and tennis and grass (*p* = 0.037) surfaces.

## 4. Discussion

We did not find a difference in median WBGTs among three different athletic surfaces during any of the three practice periods. However, microclimatic differences in dry-bulb temperature, dewpoint temperature, and wet-bulb temperature among the surfaces were observed at various times and help to explain the lack of difference in WBGT.

In the morning, we found statistically significant differences in dewpoint temperature and wet-bulb temperature but no difference in dry-bulb or globe temperatures. Grass and the underlying soil can add moisture to the air via evapotranspiration, increasing dewpoint temperatures compared with impervious surfaces, like the tennis court or artificial turf surfaces, that are designed to quickly drain away water [13,23]. The wet-bulb temperature is a function of multiple variables, including solar radiation, dry-bulb temperature, wind speed, and humidity [24]. Given no statistical difference in dry-bulb temperature and solar radiation among surfaces during this period, the greater wet-bulb temperature over grass is driven by the greater atmospheric moisture as indicated by the higher dewpoint temperature.

During midday and afternoon, we observed statistically significant differences among surfaces in dry-bulb and dewpoint temperatures. The artificial turf and tennis surfaces had greater median dry-bulb but lower dewpoint temperatures than the grass surface. The hotter dry-bulb temperature is in line with previous research and associated the greater transfers of sensible heat via convection of hotter air from the drier surfaces [13,14,15]. As in the morning, the greater dewpoint temperature over the grass field is related to the evapotranspiration of moisture into the lower atmosphere. The lack of significant difference in wet-bulb temperature is due to counteracting factors. As mentioned above, wet-bulb temperature is a function of multiple meteorological variables. Over the artificial turf and tennis surfaces, the greater dry-bulb temperature would serve to increase the wet-bulb temperature, but the lower dewpoint temperature would offset this increase. In contrast, the lower dry-bulb temperature over the grass surface would decrease the wet-bulb temperature, but this would be offset by the greater dewpoint temperature. This finding is different than observed by Kandelin et al. (1976) who observed a greater wet-bulb temperature over artificial turf when compared with a grass field [14]. An explanation for this is that the Kandelin study did not measure humidity independently over each surface but rather used one measurement. Thus, the higher wet-bulb temperatures over the artificial turf are driven by the greater air temperatures. Lastly, the globe temperature is determined by several factors including solar radiation, air temperature, and wind [24]. While the greater dry-bulb temperatures over the tennis and artificial turf surfaces may slightly increase the globe temperature, the small overall difference among surfaces is likely due to the similar solar radiation inputs experienced by the nearby study sites. In sum, given the high weight of the wet-bulb temperature (which was not different among sites) in the WBGT computation, the small differences in dry-bulb and globe temperatures did not lead to a statistically significant difference in the WBGT.

Our findings are consistent with those of Kopec (1977) who also compared WBGT among different surface types (e.g., hardcourt tennis, artificial turf, and grass) in a similar humid subtropical climate [16]. He observed only small differences in WBGT between the grassy control site and other sites, likely due to counteracting variables in the WBGT equation and the heavy weighting of the wet-bulb temperature component. For instance, a detailed case study between the grassy control site and an artificial turf field showed that the artificial turf surface had greater average dry-bulb and globe temperatures but a slightly lower wet-bulb temperature. The magnitude of the average differences in both dry-bulb (2.2 °C) and globe temperatures (3.6 °C) were greater than those found in this study, however. Possible explanations may be the differences in thermal characteristic of the artificial turf surfaces (Astroturf vs. FieldTurf), the short period of the Kopec’s case study (two hours on a single day), and the distance between sites which could influence solar radiation. In fact, Kopec (1977) hypothesized that solar radiation in response to changing cloud cover rather than surface type was the key driver of WBGT variations between sites in his study. We similarly found that changes in solar radiation were highly correlated with WBGT and resulted in large swings in values, regardless of surface, over short time periods. However, the nearness of our three sites allowed us to control for solar radiation as a factor in explaining instantaneous differences in WBGT.

In our study, we identified some limitations that may impact our findings. First, our study was performed in a humid, subtropical climate with a well-watered grass surface. Further research is needed to confirm if our findings can be more broadly applied to conditions when the grass surface may have low soil moisture, whether due to a drought or the prevailing climate (e.g., arid region), which could influence evapotranspiration and low-level moisture [25]. Second, we focused on three common athletic surface types. While further work is needed to confirm our findings over different surfaces such as rubberized track surfaces or brick dust often used with baseball and softball infields, our work is suggestive that variability in solar radiation creates larger WBGT variations within surface type than between surface type. Third, our study focused narrowly on whether WBGT varied by athletic surface type. We cannot conclude there is no difference in heat stress to athletes among athletic surfaces. In addition, our results should not be generalized to other heat indices. Other measures, such as the heat index, have different assumptions and input variables than the WBGT, which could affect whether there are meaningful differences in the index values among athletic surfaces. Finally, our results are applicable to nearby sites (less than 0.5 km). Longer distances may influence solar radiation variability between sites.

## 5. Conclusions

Our study indicates that in a humid, subtropical climate over a well-watered grass field, there is no difference in WBGT when compared to artificial turf and hardcourt tennis surfaces. Yet, there are clear microclimatic differences in dry-bulb and dewpoint temperatures among the three surfaces that provide counterbalancing influences on components of the WBGT, ultimately limiting the total difference in the index. Thus, a single monitoring site is sufficient to capture representative WBGTs over a variety of commonly used athletic surfaces in close proximity, when meeting our study conditions.

## Figures and Tables

**Table 1:** Athletic surface WBGT correlations (*n* = 242 observations) with weather variables measured at the on-site WeatherSTEM observing station. AT is artificial turf, WBGT is wet-bulb globe temperature.

	AT WBGT	Grass WBGT	Tennis WBGT
Dry-bulb Temperature	0.52	0.32	0.50
Dewpoint Temperature	0.22	0.23	0.15
Solar Radiation	0.60	0.65	0.66
